# Supplementary material for: Loss of ERβ in Aging LXRαβ Knockout Mice Leads to Colitis
Source: Int J Mol Sci. 2023 Aug 5;24(15):12461. doi: 10.3390/ijms241512461 (PMC10419301; doi:10.3390/ijms241512461)
Supplement: Supplementary file 1 [file ijms-24-12461-s001.zip › ijms-2529475-supplementary.pdf]

Supplementary Figures for

Loss of ERβ in aging LXRαβ knockout mice leads to colitis

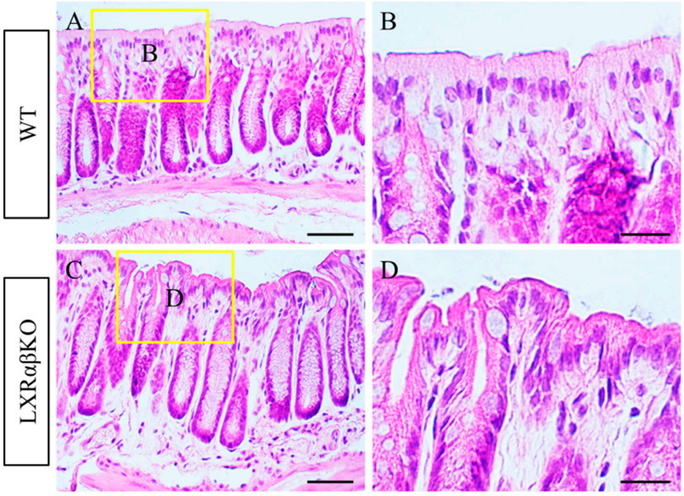

Figure S1. Morphological comparison of colon between WT and LXRαβ$^{-/-}$ littermates. Compared with WT (A and B), the colonic epithelium of LXRαβ$^{-/-}$ mice were flat, with more cells (macrophages) infiltrating the lamina propria (C and D). Scale bars: A and C, 50 μm; B and D, 20 μm.

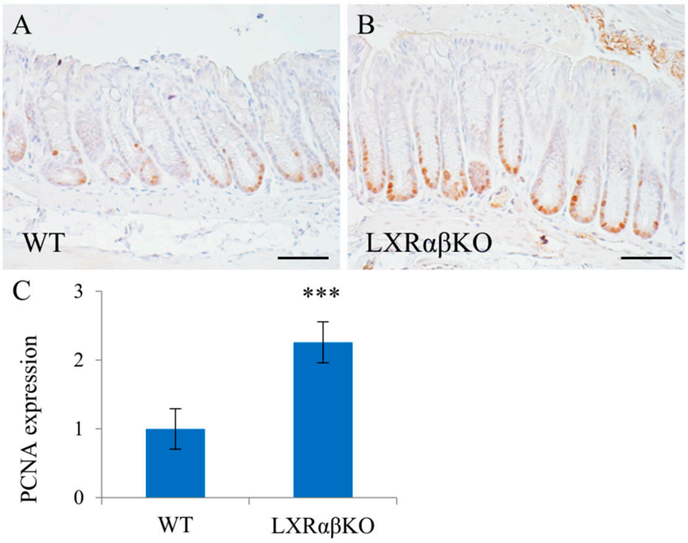

Figure S2. Proliferation levels in the colon of LXRαβ$^{-/-}$ mice. Cell proliferation (PCNA-positive) was significantly increased (***$P < 0.001$) in the colon of LXRαβ$^{-/-}$ mice compared with WT (A-C). Scale bars: A and B, 50 μm.

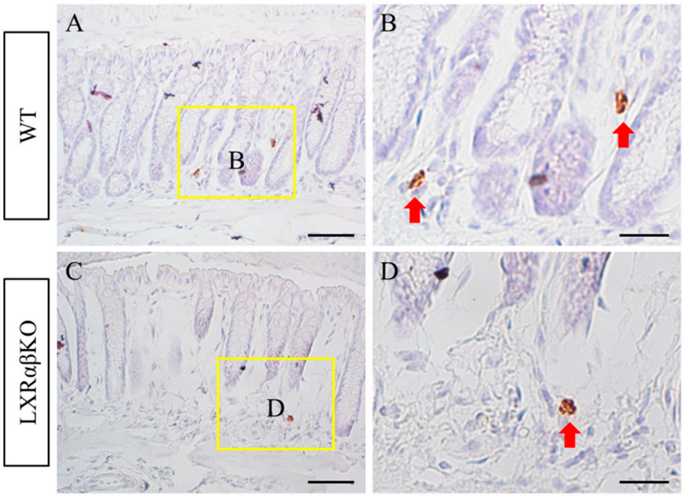

Figure S3. Neutrophil expression in the colon of LXRαβ$^{-/-}$ mice was unchanged. A small amount of MPO-positive neutrophils were expressed in the colon of WT and LXRαβ$^{-/-}$ mice, but there was no significant change (A-D). MPO: Myeloperoxidase. Red arrows indicate MPO-positive neutrophils. Scale bars: A and C, 50 μm; B and D, 20 μm.

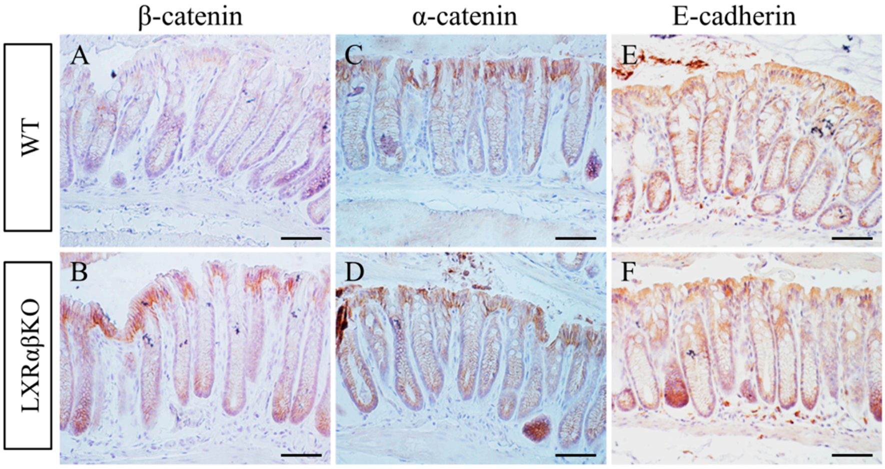

Figure S4. Expression of adherens junction molecules in the colon of WT and LXRαβ$^{-/-}$ littermates. Expression of β-catenin in the colon of WT and LXRαβ$^{-/-}$ littermates (A and B). The expression of α-catenin was not significantly changed in the colon of WT and LXRαβ$^{-/-}$ mice (C and D). No change in E-cadherin expression in the colon of WT and LXRαβ$^{-/-}$ mice (E and F). Scale bars: A-F, 50 μm.
